# Enhancement of surfactant performance via titanium dioxide nanoparticles: implication for oil recovery in sandstone

**DOI:** 10.3389/fchem.2024.1457753

**Published:** 2024-11-15

**Authors:** Miftah Hidayat, Rima Megayanti, Ndaru Cahyaningtyas, Mahruri Sanmurjana, Zeta Nur Muhammad Yahya, Adityawarman, Utjok W. R. Siagian, Taufan Marhaendrajana

**Affiliations:** ^1^ Department of Petroleum Engineering, Faculty of Mining and Petroleum Engineering, Institut Teknologi Bandung, Bandung, Indonesia; ^2^ Enhanced Oil Recovery Laboratory, Faculty of Mining and Petroleum Engineering, Institut Teknologi Bandung, Bandung, Indonesia; ^3^ Research Center for CO_2_ and Flare Gas Utilization, Institut Teknologi Bandung, Bandung, Indonesia; ^4^ Center for Research on Energy Policy, Institut Teknologi Bandung, Bandung, Indonesia

**Keywords:** surfactant, titanium dioxide nanoparticles, interfacial tension, wettability, oil recovery

## Abstract

The application of titanium dioxide nanoparticles in the petroleum research area has received ample attention in recent years owing to its impact on wettability-altering agents. Further, employing a surfactant injection to improve oil production in sandstone formations on an industrial scale has become an alternative solution, particularly for mature fields. However, the existing literature on the combination of alkyl ethoxy carboxylate (AEC) surfactant with titanium dioxide nanoparticles on the application of enhanced oil recovery in sandstone formations remains underreported. This study explores the impact of combining AEC surfactant with titanium dioxide nanoparticles on recovering trapped oil in sandstone by examining the interfacial tension, contact angle, zeta potential, and core flooding with various concentrations of added titanium dioxide nanoparticles (0, 0.01, 0.025, and 0.05 wt%) on AEC surfactant. Although the addition of 0.05 wt% TiO_2_ to AEC surfactant can significantly reduce the interfacial tension to the lowest value of 5.85 × 10^−5^ mN/m, our results show that the highest oil recovery in Berea sandstone (59.52% recovery factor) is achieved at the concentration of 0.025 wt% added TiO_2_ to AEC surfactant. We find that the stability of TiO_2_ nanoparticles on AEC surfactant plays a significant role in getting maximum oil recovery. These important findings from this study contribute to improving our understanding on the application of TiO_2_ combined with AEC surfactant to achieve more efficient and sustainable enhanced oil recovery in sandstone.

## 1 Introduction

Sandstone formations are one of the major oil reservoirs, approximately 50% of the total oil world’s reservoir (Bjørlykke and Jahren, 2015). The conventional oil recovery stages consist of primary, using natural energy from the reservoir, and usually, the oil recovery is around 20%; secondary, injecting the water into the reservoir, which resulting the recovery increased to 30%–50%; and tertiary, employing a sophisticated method to recovery the remaining trapped oil that cannot be extracted at secondary stages, which also known as enhanced oil recovery (e.g., Udoh, 2021).

There are numerous enhanced oil recovery (EOR) methods that are available in the petroleum industry, including (but not limited to) surfactant (Hakiki et al., 2015; Hou et al., 2016; Liang et al., 2021), polymer (Santoso et al., 2018; Sieberer et al., 2019; Zeynalli et al., 2023), gas injection (e.g., CO_2_, N_2_; Li et al., 2019; Hill et al., 2020), and thermal (e.g., steam flood; Chu, 1985). One of promising approach within the EOR methods is the use of surfactant, which has the ability to reduce the interfacial tension (IFT) between crude oil and natural brine (Xu et al., 2013; Zhang et al., 2022), altering the wettability of the reservoir (Hou et al., 2015; Kumar and Mandal, 2020; Yao et al., 2021) and also, forming of microemulsion to improve the mobility (Santanna et al., 2009; Hematpur et al., 2021; Mahboob et al., 2022). Among various types of surfactants, alkyl ethoxy carboxylate (AEC) surfactant has become a potential candidate due to its characteristic, which has relatively cheaper raw material to synthesize it and also this type of surfactant had been reported by literatures to stand up to wide range of salinity and high temperatures (Adkins et al., 2012; Lu et al., 2014; Jürgenson et al., 2015; Aslam et al., 2017). Moreover, a recent study by Megayanti et al. (2023) suggests that the incorporation of titanium dioxide (TiO_2_) nanoparticles can enhance the performance of AEC surfactant significantly by lowering the IFT at the lowest value of 5.85 × 10^−5^ mN/m and contact angle to the minimum value of 8.8° on a thin section of Berea sandstone with the air as the immiscible phase. The characteristic of nanoparticles that can spread into the rock surface, forming the wedge film on the oil droplet and yielding increasing structural disjoining pressure (e.g., Wasan et al., 2011; Yahya et al., 2022; Megayanti et al., 2023) and can lead to recovering the trapped oil the subsurface sandstone formations more effectively.

Kumar et al. (2022) conducted a study on the effects of graphene oxide (GO) and silica (SiO_2_) nanoparticles when combined with HPAM polymer. Both types of nanoparticles effectively reduce the IFT between oil and water phases, with GO demonstrating more effectiveness due to its amphiphilic properties. Additionally, increasing the concentration of nanoparticles further enhances IFT reduction; however, excessively high concentrations may result in stability problems (Kumar et al., 2022). A study by Lashari et al. (2023) indicates that HPAM/GO-SiO₂ nanocomposites have a considerable impact on wettability, an essential aspect of the oil removal process. They found that employing HPAM/GO-SiO₂ alters the contact angle between the fluid and the reservoir surface, enhancing the fluid’s capacity to displace oil from rock pores. This change in wettability results from the interactions between the nanoparticles and the polymer matrix, which can lower surface energy and facilitate improved oil mobility (Lashari et al., 2023). Furthermore, a previous study has demonstrated that the use of TiO₂ nanoparticles can significantly improve oil recovery, raising it from 49% to 80% (Ehtesabi et al., 2015). Cheraghian (2015) investigated the effects of combining TiO₂ with polymers, observing an increase in recovery from 10.1% to 40.4%. These results highlight the importance of TiO₂ nanoparticles for enhancing oil recovery. Therefore, in this study, we are looking for the potential combination of TiO₂ nanoparticles with AEC surfactant to further optimize the recovery of trapped oil in sandstone formations.

This study aims to explore the potential synergistic effects of combining the AEC surfactant with various concentrations of TiO_2_ nanoparticles (0–0.05 wt%) to increase oil production in sandstone formations. Several investigations are carried out, including interfacial tension measurements, zeta potential and interfacial charge measurements, improved contact angle measurements in a thin section of sandstone formations, and lastly, one-dimensional core flooding test in a sandstone sample to obtain oil recovery. Our results show that adding TiO_2_ nanoparticles in AEC surfactant can reduce the interfacial tension and alter the wetting state to a more water-wet condition, compared with natural brine, resulting in increasing oil recovery in the sandstone samples to 59.52%. In addition, the stability of TiO_2_ on AEC surfactant is an essential parameter to be considered to achieve maximum oil recovery. The outcome of this study can improve our understanding of the mechanisms of the addition of TiO_2_ nanoparticles to AEC surfactant and become an alternative method for recovering the remaining oil in sandstone formations.

## 2 Materials and methods

### 2.1 Materials

An alkyl ethoxy carboxylate (AEC) anionic surfactant was used in this study. This surfactant was manufactured in-house with cooperation between Institut Teknologi Bandung and PT. Rakhara Chemical Technology. The AEC surfactant is composed of a carboxylate molecule as the polar (hydrophilic) head that has a negative charge, while the non-polar (hydrophobic) tail consists of linear alcohol of the C_10_–C_14_ carbon chains (Herawati et al., 2022; Megayanti et al., 2023). The detailed properties of the AEC surfactant can be seen in Table 1.

**TABLE 1:** The properties of the AEC anionic surfactant used herein.

Formula	Physical state	Chemical structure
C_28_H_56_O_16_	Liquid	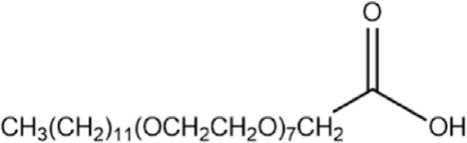

Crude oil was sourced from one of oil field in Indonesia that has a SARA (saturate, aromatic, resin, and asphaltene) analysis presented in Table 2. The SARA analysis shows that the oil sample can be categorized as light oil, dominantly consisting of saturated fraction, followed by aromatics fraction and a small fraction of resins and asphaltenes.

**TABLE 2:** The properties of the crude oil used herein (Swadesi et al., 2015).

Oil characteristics	Value
SARA	
Saturated	71.60%
Aromatics	25.49%
Resins	2.14%
Asphaltenes	0.78%
EACN (Equipment Alkane Carbon Number)	8.29
TAN (Total Acid Number)	1.23 mg KHO/g
Viscosity	0.90 cP (66°C)
API Gravity	43.45

**FIGURE 1:**
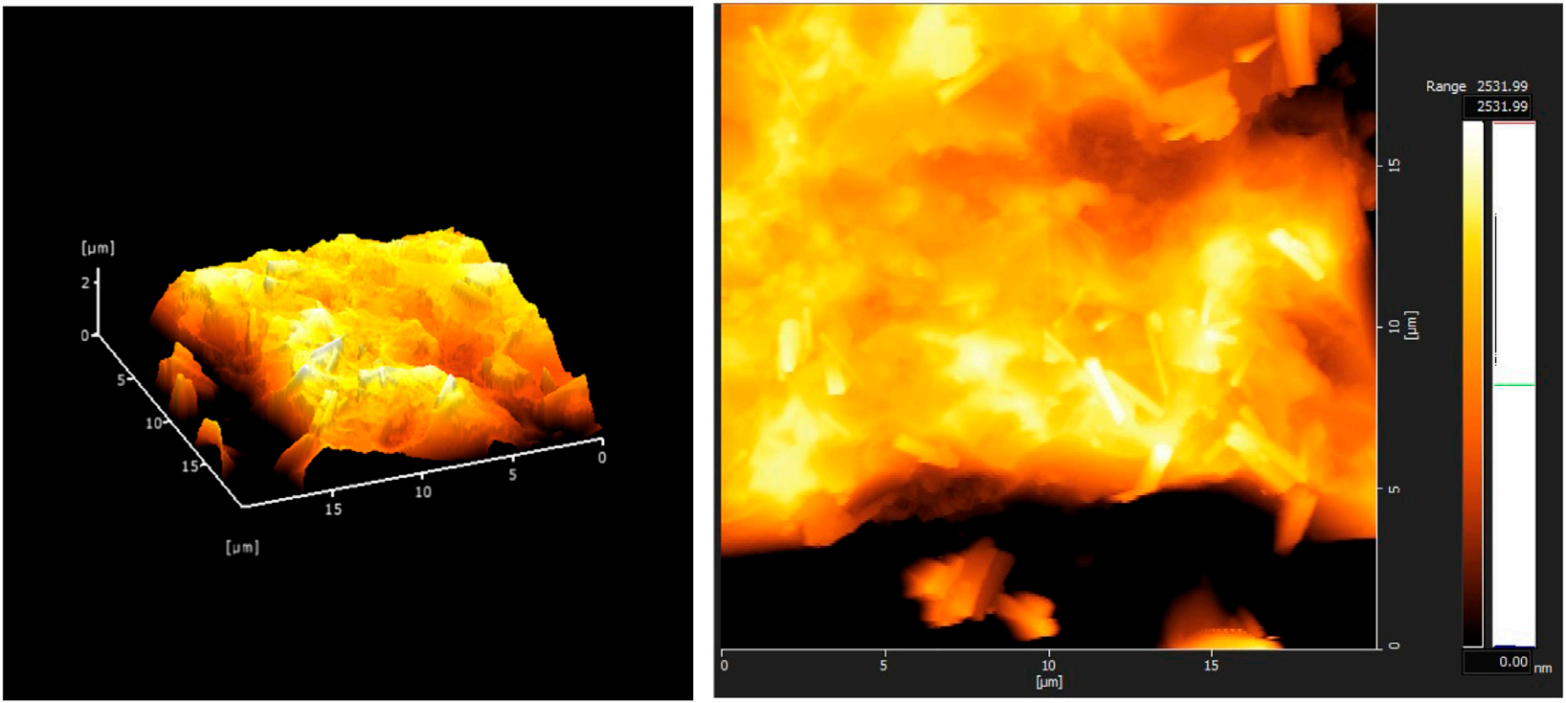
Atomic force microscopy images of Berea sandstone, RMS = 7.227 × 10^2^ nm.

In order to simulate the application of TiO_2_ nanoparticles combined with AEC surfactant in sandstone formations, Berea sandstones were selected as the representative of subsurface rock formations. A thin section of Berea sandstone was prepared by cutting a slice of approximately 1–2 mm thickness from the rock sample and mounting it onto an object glass with epoxy resin. The thin section was then air-dried in an oven for at least 3 h at 30°C. After drying, the surface of the rock was polished with 600-grit sandpaper to achieve a smooth and even texture. To characterize the surface roughness of a thin section of Berea sandstone for contact angle measurements, the thin section was taken into an automatic force microscope (AFM) supplied by Hitachi AFM5300E with a manual stage XY ± 2.5 mm and a scan range of 20 μm × 20 μm × 15 μm. The root-mean-square (RMS) surface roughness measurement was RMS = 7.227 × 10^2^ nm (see Figure 1 for the 2D and 3D topography AFM measurement results). AFM is a powerful imaging technique used to obtain high-resolution images of surface characteristics at the nanoscale. Surface roughness affects the wettability, as demonstrated by considerable changes in contact angles. However, the relationship between contact angle and surface roughness is not consistent (Aboushanab et al., 2024). Hence, to deal with roughness, polishing the rock surface is a common practice to ensure a smooth surface as we did in the preparation stage.

To determine the mineral composition of the Berea sandstone, the offcut from the rock sample was smashed into fine particles and measured using X-ray diffraction (XRD) analysis supplied by Rigaku SmartLab, Japan. The result of the XRD analysis is shown in Table 3. A brine solution with 0.8 wt% NaCl concentration was prepared to simulate the natural formation water by dissolving a single NaCl salt (purity ≥99 wt%, Sigma Aldrich) in the demineralized water. The titanium dioxide (TiO_2_) nanoparticles (purity ≥99 wt%) were obtained from XFNANO Material Tech Co., Ltd., China, and the internal structure of TiO_2_ nanoparticles has been characterized in the previous work as the spherical shape (Megayanti et al., 2023). The TiO_2_ nanoparticles were later used to formulate various solutions of 1.25 wt% AEC surfactant combined with different concentrations of TiO_2_ nanoparticles (0, 0.01, 0.025, and 0.05 wt%) for interfacial tension measurements, contact angle measurements, zeta potential investigations, and one-dimensional displacement core flooding tests. To ensure the combined solutions were uniformly dispersed, the tested solutions were formulated via ultrasonication, following the reported procedure of Megayanti et al. (2023).

**TABLE 3:** Petrophysical properties of Berea sandstone and the injection scenario of core flooding test in this study.

Berea sandstone	Injection scenario	Liquid permeability (mD)	Pore volume (mL)	Core dimension (cm)	Initial oil saturation (%)	Mineralogy
Berea #1	5 PV waterflood natural brine	134.48	3.79	Diameter: 2.54	42.23	Quartz = 93.81 wt% Feldspar = 2.29 wt% Pyrite = 2.11 wt% Clay (kaolinite and illite) = 1.79 wt%
				Length: 4.04		
Berea #2	5 PV AEC Surfactant 1.25 wt%	69.029	3.86	Diameter: 2.54	64.73	
				Length: 4.00		
Berea #3	5 PV AEC Surfactant 1.25 wt% + 0.01 wt% TiO_2_	100.701	3.93	Diameter: 2.54	53.39	
				Length: 4.00		
Berea #4	5 PV AEC Surfactant 1.25 wt% + 0.025 wt% TiO_2_	52.759	3.95	Diameter: 2.54	53.08	
				Length: 4.00		
Berea #5	5 PV AEC Surfactant 1.25 wt% + 0.05 wt% TiO_2_	109.468	3.92	Diameter: 2.54	56.04	
				Length: 4.00		

### 2.2 Methods

#### 2.2.1 Interfacial tension measurement

A spinning drop tensiometer (TX500D, USA) with an accuracy of ±3 RPM and ±0.5°C was employed to measure the interfacial tension (IFT) of crude oil and water (AEC surfactant/TiO_2_ nanoparticles). Prior to doing the measurements, the capillary tube was thoroughly cleaned using the reported procedure by Megayanti et al. (2023). The total time during the measurement was 30 min, and the measurement was conducted at a constant speed of 3000 RPM (rotation per minute). Three different temperatures, including 25°C, 40°C, and 68.3°C, were tested to assess how temperature variations have an effect on critical micelle concentration (CMC) of AEC surfactant, with the range of 0.5–2.0 wt% of AEC surfactant concentrations. Note that a temperature of 68.3°C corresponded to the actual reservoir temperature where the crude oil sample was sourced. Furthermore, we also investigated the effect of the addition of TiO_2_ nanoparticles (from 0–0.05 wt%) into AEC surfactant solution at the concentration of CMC focusing on the actual reservoir temperature (68.3°C).

#### 2.2.2 Contact angle measurement

**FIGURE 2:**
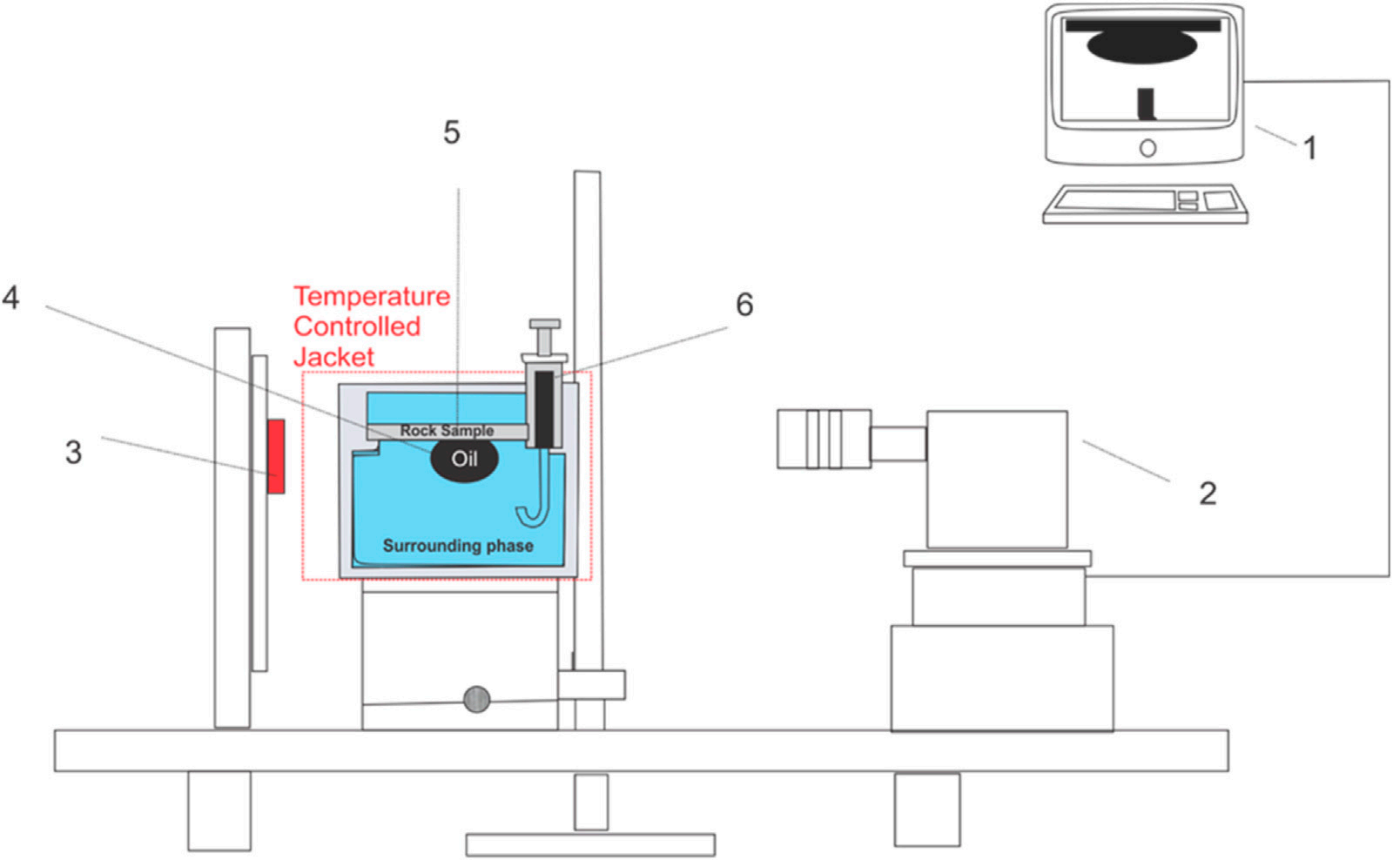
The schematic of the experimental setup for contact angle measurement (#1) Data acquisition with One Attention and ImageJ software, (#2) camera, (#3) light source (#4) oil droplet, (#5) a thin section of Berea sandstone (#6) micro syringe.

To characterize the wettability of various nanofluid concentrations combined with AEC surfactant, the sessile drop method was employed to measure the static contact angle using Theta Lite Optical Tensiometer (Accuracy of ±0.1°, Biolin Scientific), as schematically shown in Figure 2. Prior to measuring the contact angle, 140 mL of the tested solution was placed inside the sample holder. Subsequently, the thin section of the Berea sandstone (1–2 mm thickness) was put at the top of the tested solution (Figure 2). 5 mL of crude oil was placed in the micro syringe. To ensure a constant temperature condition, the temperature in the measurement cell was maintained at 25 ± 0.2°C using a temperature-controlled jacket for at least 2 hours. The measurement was begun by introducing the crude oil from the micro syringe into the thin section of the Berea sandstone. The volume of the oil droplet was 6 ∼ 7 ± 1 µL. The whole process was recorded, and the obtained image was analyzed using ImageJ software. Contact angle measurement was repeated at least three times to ensure experimental repeatability and uncertainty of the measurements.

#### 2.2.3 Zeta potential and interfacial charge measurement

Zeta potential is a physicochemical property that represents the electrostatic interaction between electrolyte/brine/water with other dispersed phases/non-aqueous phase fluid (e.g., rock mineral, crude oil, nanoparticles) at the interface (e.g., Hidayat et al., 2022a; Hidayat et al., 2022b; Vinogradov et al., 2021). In order to determine the stability of TiO_2_ nanoparticles in AEC surfactant solution, zeta potential measurement using electrophoretic mobility method was employed. The electrophoretic mobility method (EPM) relies on the relative motion between the dispersed phase and stationary water due to the applied electrical field (Delgado et al., 2007). A Horiba SZ 100 (supplied by HORIBA Scientific Co., Ltd., Japan, with an accuracy of ±2%) was used to determine the zeta potential using EPM of various nanofluid concentrations combined with the AEC surfactant solution. Further, the zeta potential of crude oil-brine, crude oil-AEC surfactant, rock mineral-brine, and rock mineral-AEC surfactant at the interfaces were also measured with the ratio between the electrolyte solution (brine/AEC surfactant) and the dispersed phase (crude oil and powdered rock sample) was 4:1 ratio. The measurement was conducted at room temperature (25 ± 0.2°C) and repeated at least three times to ensure consistent results.

#### 2.2.4 One-dimensional displacement core flooding test

**FIGURE 3:**
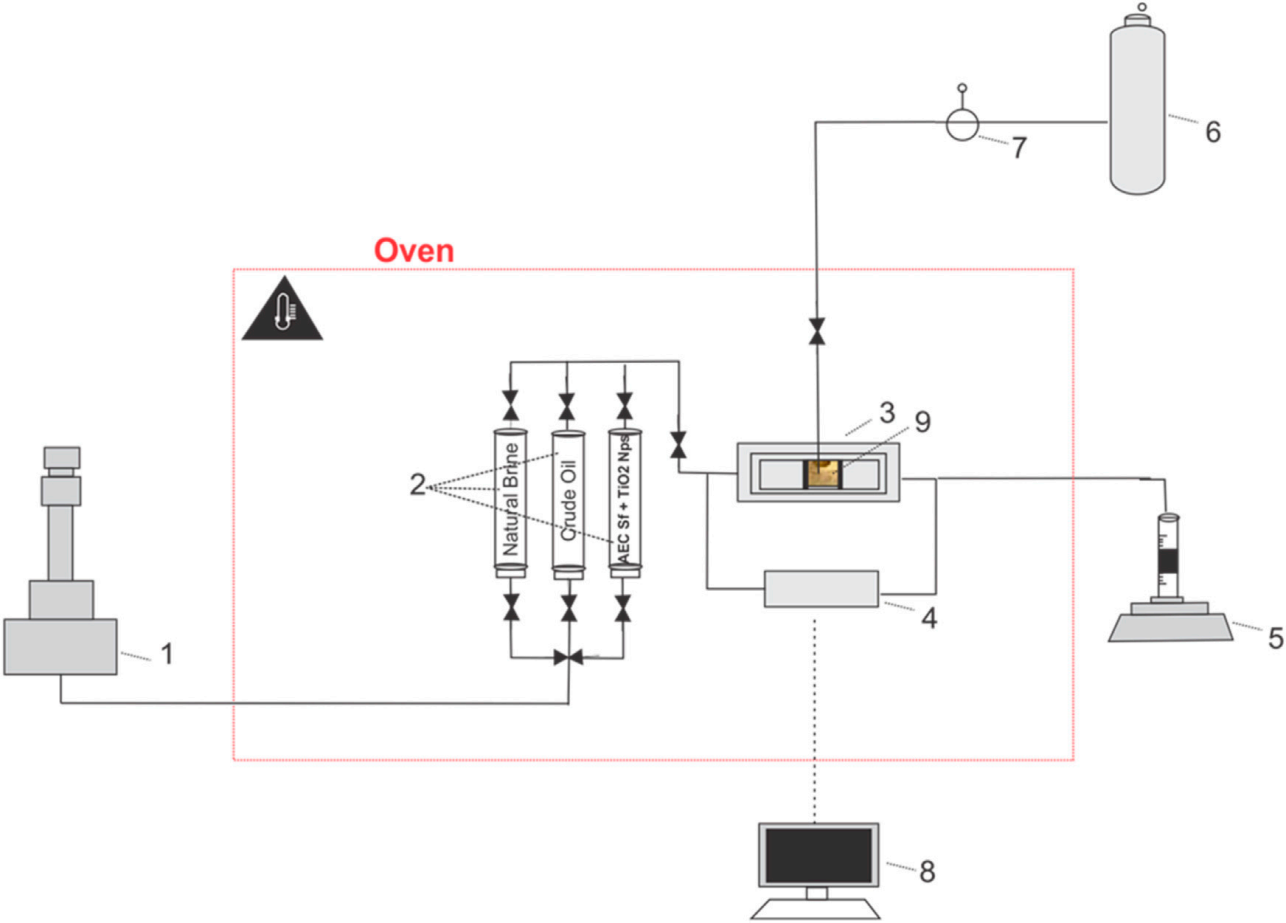
The experimental set-up was used for the core flood test. (#1) A nitronic 500D ISCO syringe pump, (#2) accumulator, (#3) a stainless-steel core holder; (#4) high precision ESI pressure transducer; (#5) measuring glass, (#6) N_2_ Cylinder, (#7) analog pressure reader for confining pressure, (#8) data acquisition, (#9) Berea sandstone sample.

Five (5) scenarios of the core flooding test (Table 3) were performed in a stainless-steel core holder cell inside the oven, allowing the core sample to be tested at reservoir temperature. A schematic of the core flooding setup is shown in Figure 3. Prior to doing the core flooding test, the dry weight of the Berea core sample was first measured using an analytical balance, and then it was saturated with natural brine (0.8 wt% NaCl) using a vacuum method for at least 24 h to ensure a fully saturated condition. The saturated core sample was weighted again to measure its wet weight, which was later used to determine the core sample’s pore volume (PV). Subsequently, the core sample was placed inside the core holder, and 200 psi of N_2_ gas was introduced into the core holder to create confining pressure. The core holder was positioned horizontally to reduce the impact of gravity. Natural brine that was placed in the accumulator was injected into the core sample using a nitronic Teledyne ISCO 500D syringe pump for at least four different flow rates until stabilized pressure differences were reached to determine the liquid permeability at room temperature conditions. The pressure difference across the core sample was recorded using high-precision ESI pressure transducer model GD4200 (accuracy of 0.15%, supplied by Esi Technology, UK). The quality of regression’s linearity (R^2^) of the plotted flow rates against the pressure differences for all measurements was confirmed to be greater than 0.99. After the preparation stage, the oven was heated to the tested reservoir temperature (68.3°C) and waited for at least 4 h to ensure constant temperature conditions.

The next step was oil saturation, which involved injecting crude oil into the core sample by applying a constant injection rate of 0.3 mL/min. The injection stage was maintained until the residual water saturation was achieved, indicating no effluent water was observed in the measurement glass. In order for the fluids inside the core sample to be evenly distributed, the core sample was aged for 24 h at reservoir temperature. Further, the tested solutions were injected into the core sample using a constant injection rate of 0.3 mL/min for five (5) pore volumes. The oil produced from the core sample was recorded to determine the recovery. After each scenario, the core holder, flow line, and accumulator were thoroughly cleaned using solvent and demineralized water before being used for the next core flooding experiment.

## 3 Results and discussion

### 3.1 Effect of TiO_2_ nanoparticles and AEC surfactant on the interfacial tension

**FIGURE 4:**
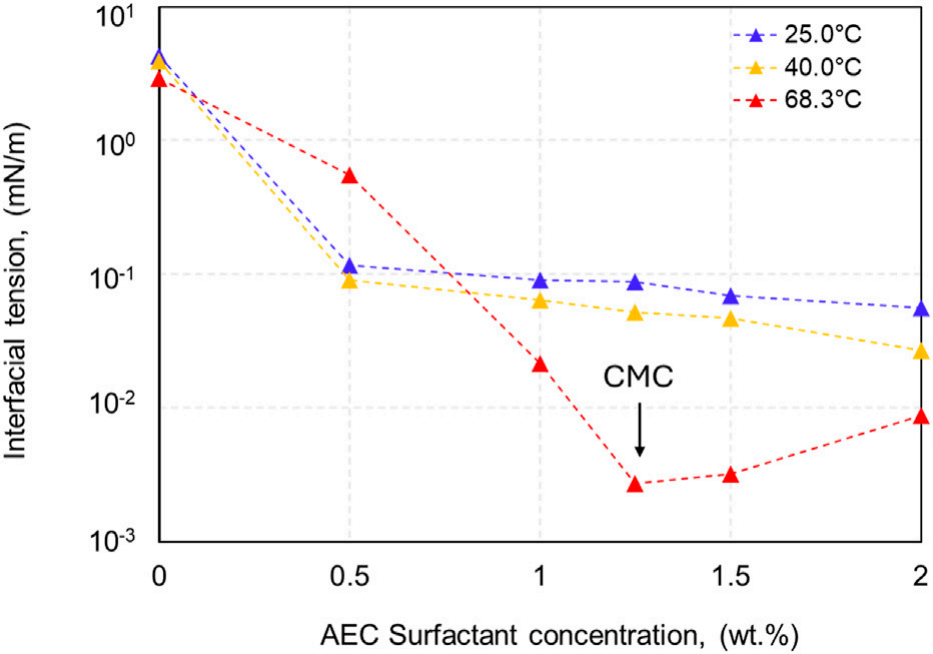
The interfacial tension of the oil-water system with AEC surfactant concentrations in range of 0.5–2 wt% at various temperatures.

Various concentrations of AEC surfactant ranging from 0.5 to 2.0 wt% were evaluated to identify the surfactant’s critical micelle concentration (CMC) at various temperatures (25°C, 40°C, and 68.3°C), as demonstrated in Figure 4. The temperatures of 25°C and 40°C exhibit similar characteristics, both showing a CMC of AEC surfactant happened at the concentration above 2.0 wt%, as the measured interfacial tension is still decreasing with increased concentration of AEC surfactant. We limit our tested concentration to a maximum of 2.0 wt% due to the consideration that high surfactant concentration has a very small opportunity to be applied in the field application. Furthermore, at the temperature of 68.3°C, the CMC of AEC surfactant occurred at the concentration of 1.25 wt%. Comparing the CMC point of each temperature, we observed that with increasing temperature, the CMC point decreases from >2.0 wt% at the temperature of 25°C and 40°C to become 1.25 wt% at a temperature of 68.3°C. We argue that as the temperature increases, the water molecules in the surfactant decrease due to the destruction of hydrogen bonds and yield to increased hydrophobic interaction (Kang et al., 2001), therefore forming the CMC at lower concentration.

**FIGURE 5:**
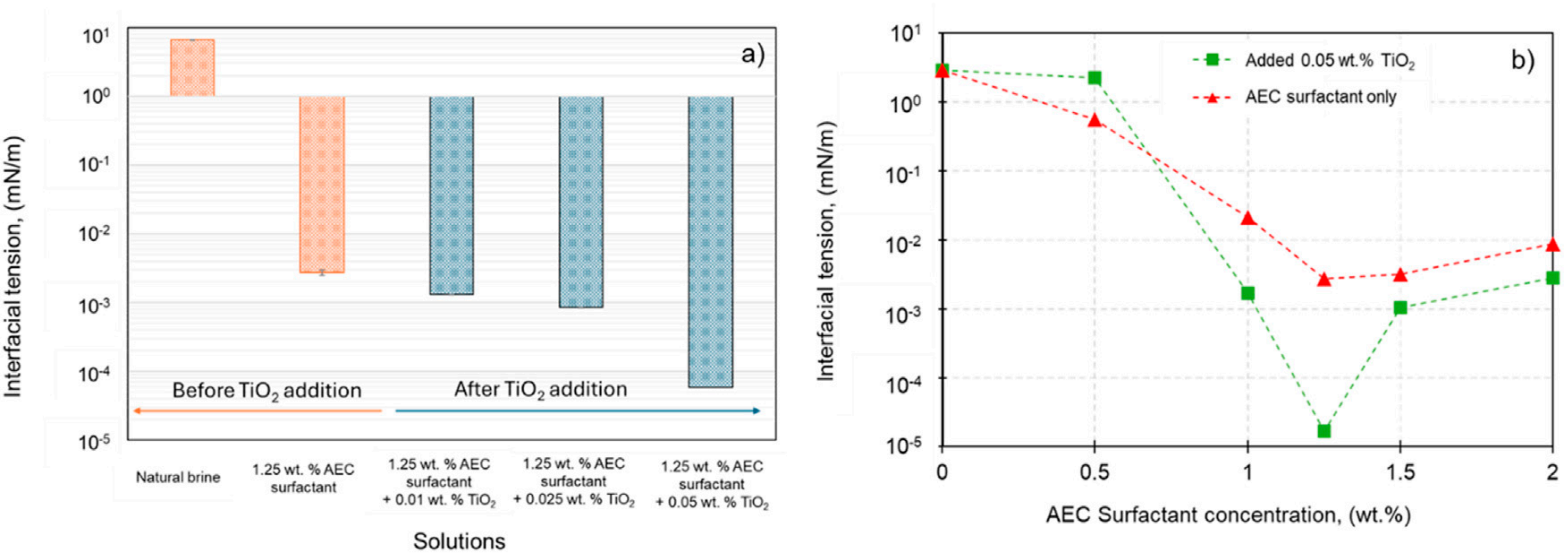
**(A)** The interfacial tension between crude oil and natural brine in combination with AEC surfactant and TiO_2_ nanoparticles (adapted from Megayanti et al. (2023)). **(B)** The interfacial tension of various concentrations of AEC surfactant (0.5–2.0 wt%) before and after the addition of 0.05 wt% TiO_2_ nanoparticles.

In order to investigate the effect of additional TiO_2_ nanoparticles and AEC surfactant on the IFT, different concentrations of TiO_2_ ranging from 0 wt% to 0.05 wt% are added into AEC surfactant with 1.25 wt% concentration, as illustrated in Figure 5A, corresponding to the CMC point at 68.3°C. In this stage, the experimental temperature is set to 68.3°C, similar to the actual condition of reservoir temperature. From Figure 5A, adding TiO₂ nanoparticles into AEC surfactant significantly improves the surfactant’s performance by reducing interfacial tension by nearly two orders of magnitude. We propose that this effect arises from the interaction between TiO₂ nanoparticles and AEC surfactant molecules, where the surfactants adsorb onto the nanoparticles via electrostatic interactions. Due to their small size, the TiO₂ nanoparticles migrate to the interface, causing some surfactant molecules to desorb from the nanoparticles and reposition at the interface, further lowering the IFT (Xu et al., 2022). Our results align with Fereidooni Moghadam and Azizian (2014) findings, confirming that our observation is not due to experimental error.

Furthermore, we extend our investigation to see the effect of TiO_2_ nanoparticles on the CMC point of AEC surfactant. Several concentrations of AEC surfactant (0–2.0 wt%) were combined with fixed 0.05 wt% concentration of TiO_2_, as demonstrated in Figure 5B. The 0.05 wt% concentration of TiO_2_ was selected due to its ability to reduce the IFT to the lowest value of 5.85 × 10^−5^ mN/m combined with 1.25 wt% AEC surfactant. Figure 5B shows the CMC of AEC surfactant after TiO_2_ addition still occurred at a concentration of 1.25 wt%, similar with CMC of AEC surfactant only. Moreover, the IFT after the addition of 0.05 wt% TiO_2_ on AEC surfactant at the concentration 1.0–2.0 wt% is smaller than using surfactant only, except for the concentration of 0.5 wt%. We argue that in a smaller concentration of surfactant (0.5 wt%) combined with the addition of 0.05 wt%. TiO_2_ does not significantly reduce the IFT. This happened because the mechanism of adsorption and desorption of AEC surfactant molecules onto nanoparticles on a small amount of surfactant concentration is less effective compared with the condition in higher AEC surfactant concentrations (1.0–2.0 wt%). However, further experimental programs combined with molecular dynamic simulation are needed to confirm our hypothesis.

### 3.2 Effect of TiO_2_ nanoparticles and AEC surfactant on zeta potential

**FIGURE 6:**
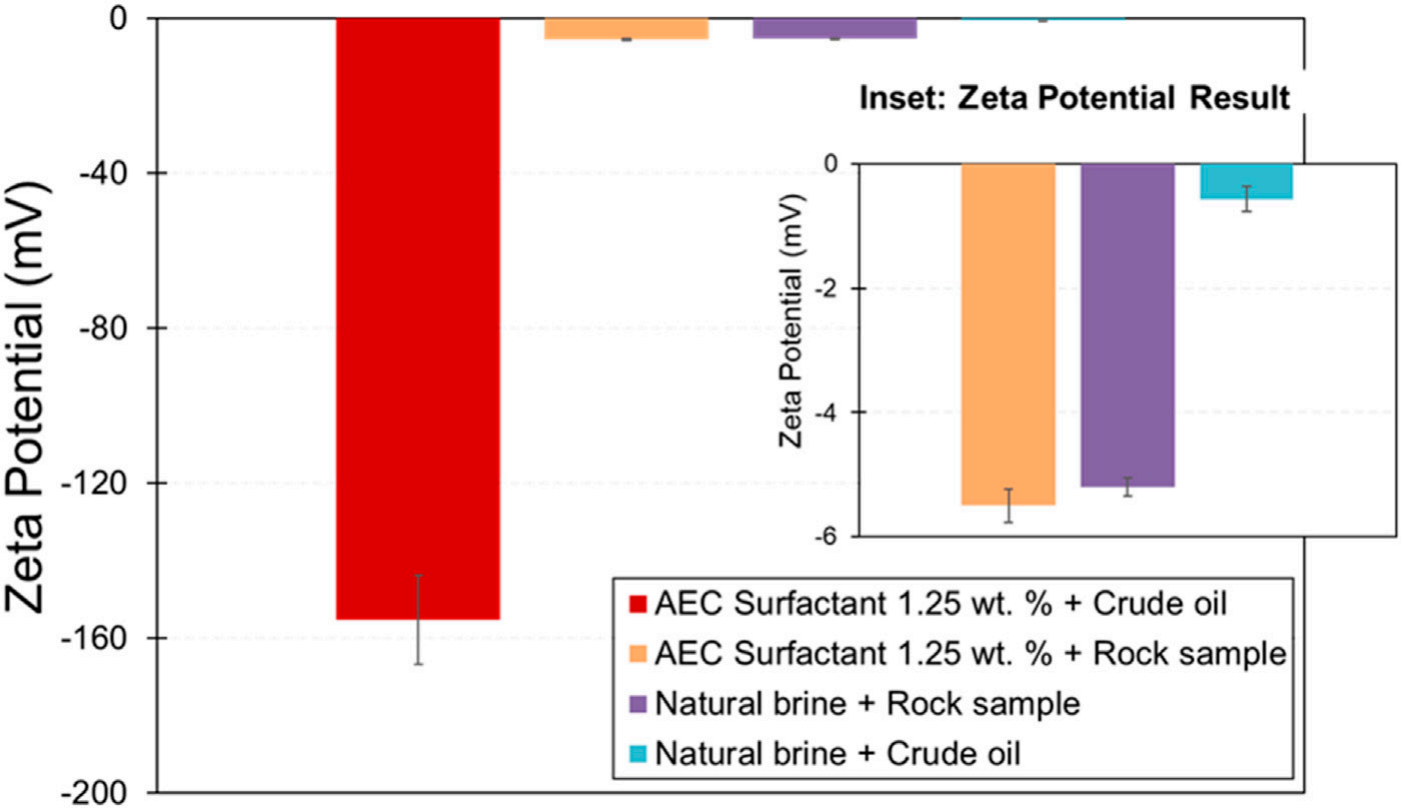
Zeta potential of crude oil-natural brine/AEC surfactant and rock mineral-crude oil-natural brine/AEC surfactant. The error bars represent the standard deviation of experimental uncertainty.

The zeta potential of crude oil-natural brine (−0.57 mV) and rock mineral-natural brine (−5.20 mV) is relatively small negative (Figure 6), which represents the interfacial charge of oil-brine and rock-brine is negatively charged and suggesting weak electrostatic repulsion between the two interfaces and yield to partially or unstable water film (Jackson et al., 2016). The polarity of the zeta potential rock-brine interface measured in this study is also consistent with the previous studies by Walker and Glover, 2018; Li et al., 2018 on Berea sandstone, confirming that the zeta potential of Berea sandstone in NaCl brine is negative.

On the other hand, the magnitude zeta potential of crude oil-AEC surfactant (−155.27 mV) and rock mineral-AEC surfactant (−5.50 mV) has increased compared with respective natural brine cases due to pH and surfactant concentration. Similar behavior is also observed in the previous study by Awan et al. (2022), by measuring the zeta potential of anionic surfactant solution in a rock sample, resulting in the magnitude of zeta potential being more negative. In addition, Hou et al., 2018 suggests that the zeta potential of aged quartz powder in anionic surfactant becomes more negative with increasing surfactant concentration and then stable at constant value. We argue that the negative polar head of anionic surfactant is responsible for changing the zeta potential to be more negative by electrostatically attracted into the rock surface, which extends the thickness of the electrical double layer. We suggest the mechanism responsible for this phenomenon is due to the ion-pair mechanism on the head of the anionic surfactant with rock surface, similar to the case presented by Hou et al., 2018. However, to confirm this argument, additional experiments related to the zeta potential of anionic surfactants with the presence of different rock minerals and crude oil, complementing with a molecular dynamic simulation study, are needed to be carried out in the near future.

**FIGURE 7:**
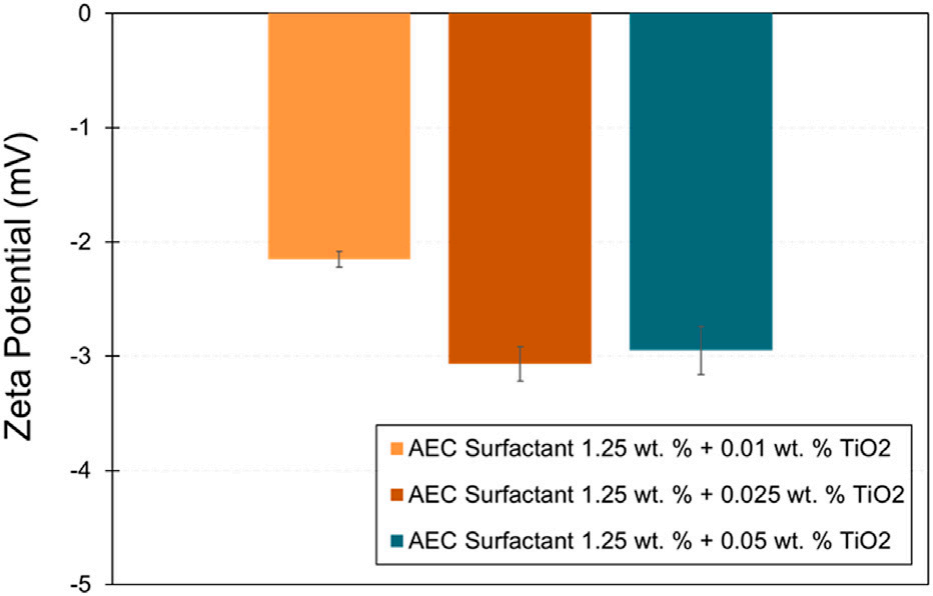
Zeta potential of various added concentrations TiO_2_ nanoparticles on AEC surfactant. The error bars were obtained from the standard deviation of measurement repeatability.

Figure 7 shows the zeta potential of AEC surfactant with various added concentrations of TiO_2_ nanoparticles (0.01, 0.025, and 0.05 wt%). The zeta potential becomes more negative with increasing the concentration of TiO_2_ nanoparticles. Note that the zeta potential at a concentration of 0.05 wt% TiO_2_ is slightly smaller than with 0.025 wt% TiO_2_, within the experimental uncertainty. Therefore, the addition of TiO_2_ at a concentration of 0.025 and 0.05 wt% may not change the zeta potential significantly. In terms of the magnitude of zeta potential on various added concentrations of TiO_2_ nanoparticles, small magnitude zeta potential indicates that the stability of nanofluids is electrically more unstable and tends to precipitate (El-Sayed et al., 2012; Al-Anssari et al., 2017). Consequently, AEC surfactant with a concentration of 0.025 wt% TiO_2_ is relatively more stable than 0.01 and 0.05 wt% TiO_2_.

### 3.3 Effect of TiO_2_ nanoparticles and AEC surfactant on contact angle

**FIGURE 8:**
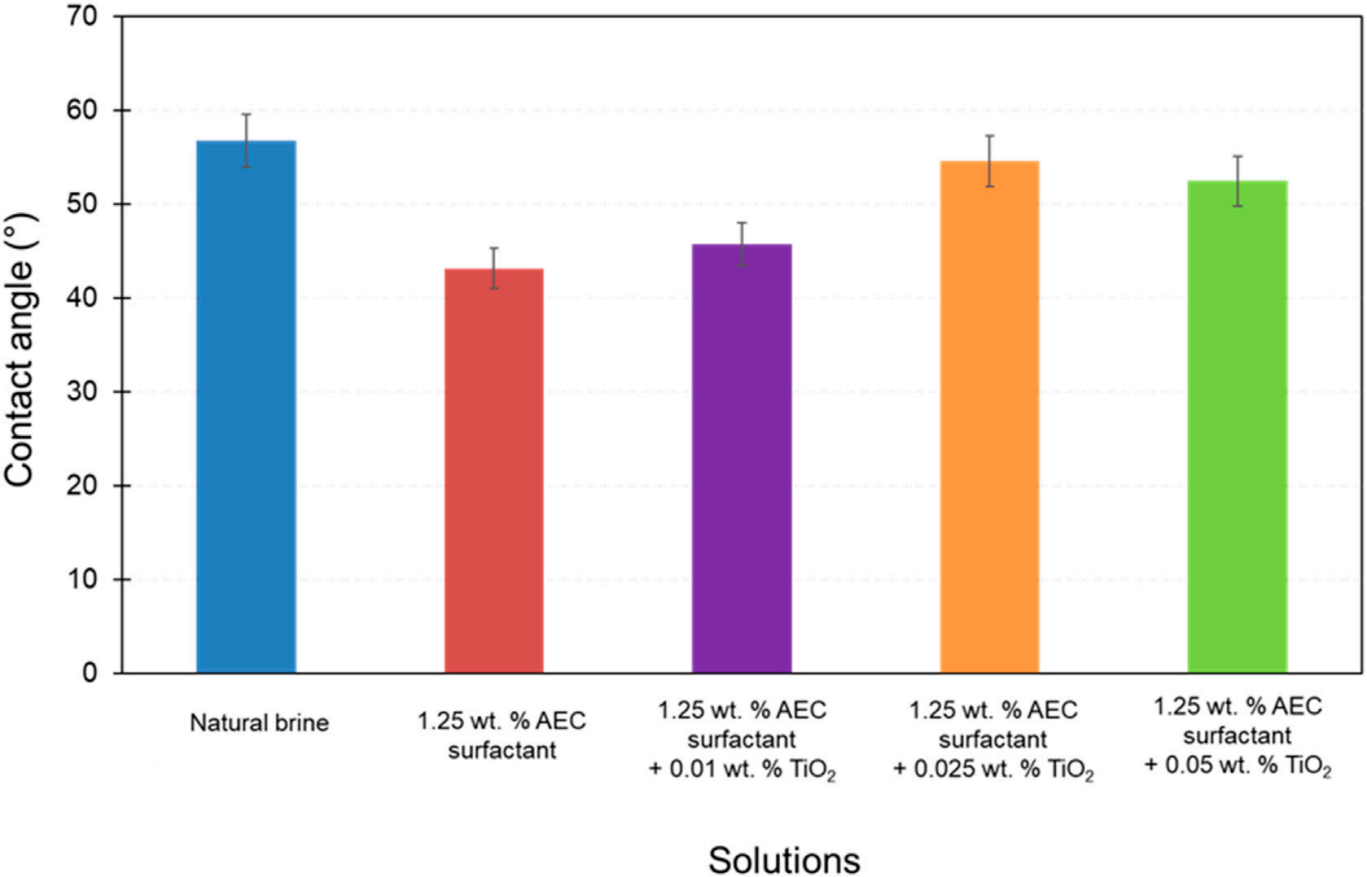
The contact angle of all tested solutions on a thin section of Berea sandstone. The error bars denote the standard deviation of the measurements.

A previous study by Megayanti et al. (2023), measured the contact angle of TiO_2_ nanoparticles combined with AEC surfactant on a thin section of Berea sandstone by considering the immiscible phase is air (see Figure 8 on Megayanti et al. (2023)). In this study, the contact angle measurement was improved by introducing oil droplets inside the water phase (e.g., natural brine, AEC surfactant, TiO_2_ nanoparticles) in order to replicate the actual subsurface condition where only crude oil and water exist. Therefore, this measurement was essential to understand the effects of TiO_2_ nanoparticles and AEC surfactant to alter the wettability from its initial condition, which are demonstrated in Figure 8. The highest measured contact angle is obtained on natural brine (56.8°), implying the current wetting state is moderately water-wet (Fanchi, 2018). On the other hand, the lowest measured contact angle is achieved by AEC surfactant 1.25 wt% (43.2°), indicating that AEC surfactant 1.25 wt% has the ability to change the surface wettability to become more water-wet condition. Furthermore, the contact angle increases with the added concentration of TiO_2_ nanoparticles on AEC surfactant, from 45.8° at 0.01 wt% to 54.6° at 0.025 wt%, and then decreases when the added concentration of TiO_2_ becomes 0.05 wt% (52.5°), within the experimental uncertainty.

The TiO_2_ nanoparticles can interact with AEC surfactant molecules and the surface of Berea sandstone. Generally, the addition of nanoparticles shifted the wettability to be more water-wet (Hendraningrat and Torsæter, 2014; Nazari Moghaddam et al., 2015). However, in this study, we observed the opposite trend as the contact angle increases with increasing the concentration of TiO_2_ nanoparticles on AEC surfactant within the experimental uncertainty. We attributed this condition to the stability of TiO_2_ nanoparticles on AEC surfactant being relatively low, which we can also confirm from the zeta potential value of AEC surfactant with TiO_2_ ranging from −2.0 to −3.0 mV (Figure 7). Hence, the impact of TiO2 nanoparticles to shift the wettability to be more water-wet by creating a wedge film to increase the disjoining pressure is less effective. Note that the contact angle of various TiO_2_ nanoparticles with AEC surfactant is still smaller than with natural brine only, suggesting the addition of TiO_2_ nanoparticles on AEC surfactant can alter the wettability to be more water wet.

### 3.4 One-dimensional core flooding test

**FIGURE 9:**
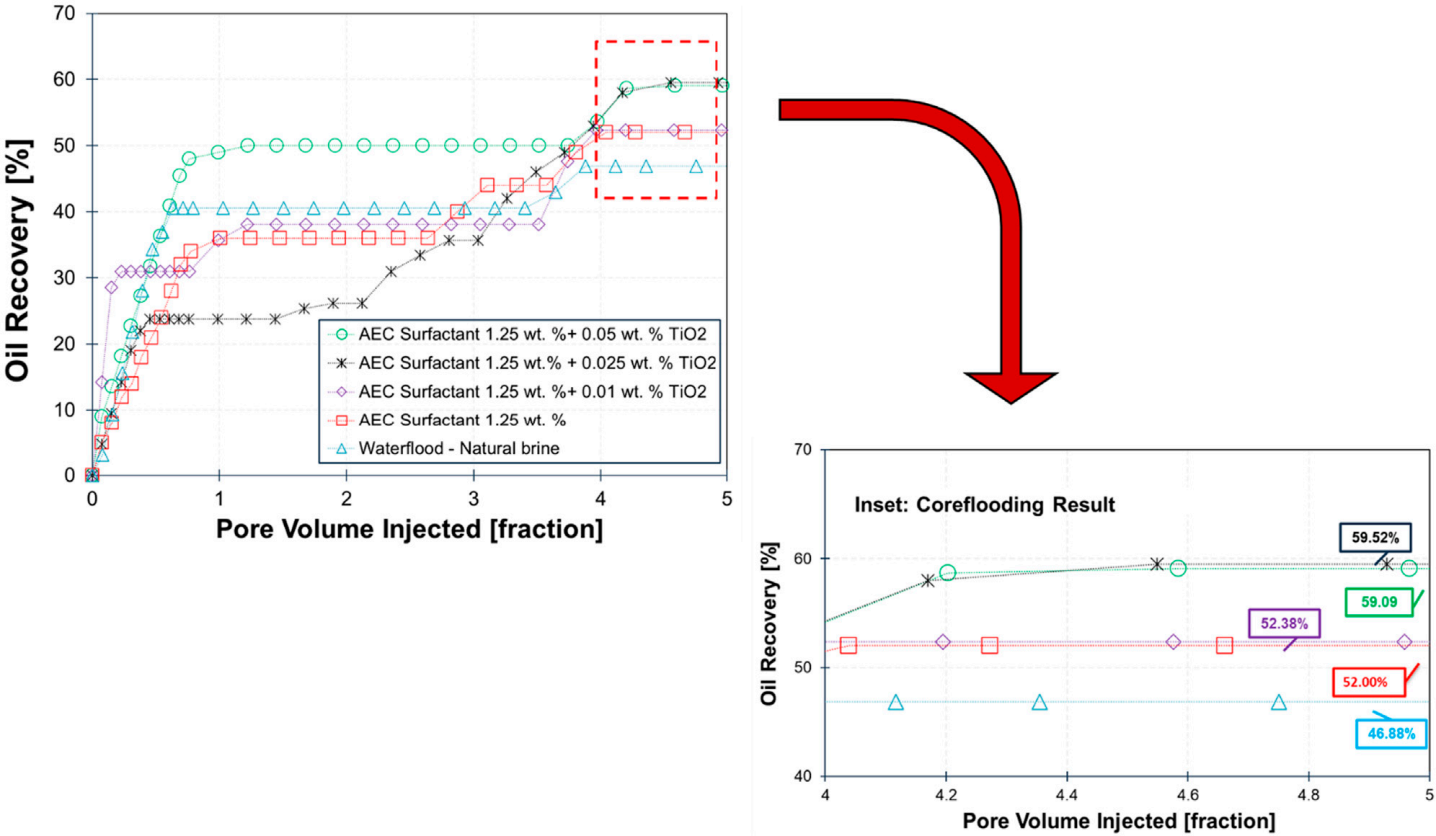
Summary of core flooding results of five different scenarios on Berea sandstone.

The result of core flooding for five scenarios on Berea sandstone is demonstrated in Figure 9. The lowest oil recovery is obtained by waterflooding of 0.8 wt% NaCl natural brine (recovery factor, RF: 46.88%). When the injected fluid is changed into AEC surfactant 1.25 wt%, the total recovery of oil is observed to be higher than waterflood (RF: 52%). The increased oil production is attributed to the reduction of interfacial tension between crude oil and brine by three orders of magnitude (see Figure 4 related the IFT in 68.3°C from 0 wt%; natural brine to 1.25 wt% AEC surfactant), and the wettability alteration also plays an essential role as the water contact angle decreased from 56.8° to 43.2° using natural brine and AEC surfactant, respectively (see Figure 8). In addition, the electrostatic repulsion of AEC surfactant-crude oil and AEC surfactant-rock has increased significantly (Figure 6) and yields, forming a stable water film and resulting in an increase in oil recovery.

Furthermore, the highest oil recovery is achieved by injection of AEC surfactant combined with 0.025 wt% TiO_2_ nanoparticles (RF: 59.52%), which increased significantly from waterflood and AEC surfactant flooding. The interfacial tension of this combined solution with crude oil is 8.50 × 10^−4^ mN/m, which can be categorized as ultra-low interfacial tension. However, the contact angle of this combined solution is 54.6°, only slightly lower than using natural brine. Therefore, we argue that reducing interfacial tension is more dominant in increasing oil production for this scenario. In addition, the zeta potential of 0.025 wt% TiO_2_ nanoparticles on AEC surfactant has the highest magnitude compared to other solutions (0.01 and 0.05 wt%), indicating that this combined solution is more stable than others.

Moreover, the oil recovery from the injection of AEC surfactant with 0.05 wt% TiO_2_ nanoparticles is 59.09%, slightly lower than with 0.025 wt% TiO_2_. On the other hand, the interfacial tension of this combination has the lowest value (5.85 × 10^−5^ mN/m) compared with all tested solutions, with contact angle on a thin section of Berea sandstone corresponding to 52.5° (slightly lower than 0.025 wt% TiO_2_). Hence, the oil recovery of this combined solution is expected to give the highest oil recovery. We hypothesize that due to the stability of 0.05 wt% TiO_2_ nanoparticles on AEC surfactant being less stable than 0.025 wt%, the TiO_2_ nanoparticles are easier to precipitate and yield to reducing the performance of AEC surfactant with 0.05 wt% TiO_2_ nanoparticles and resulting in an inefficient displacement of trapped oil during core flooding test. Therefore, we argue that the stability of TiO_2_ nanoparticles on AEC surfactant plays an essential role in making the combined solution reach the optimum performance to mobilize the trapped oil in sandstone samples.

Furthermore, we also observe that the response oil recovery for each scenario of core flooding is different and unique. For example, at the added concentration of 0.025 wt% TiO_2_ combined with 1.25 wt% AEC surfactant, the oil recovery is low until 2 pore volume injections and, after that, is increased significantly. We attributed this behavior to the drainage and imbibition processes during core flooding tests are influenced by several factors, including heterogeneity, wettability, and rock properties, particularly permeability. In low-permeability rock samples, these processes take more time compared to high-permeability samples (Hendraningrat et al., 2013). As a result, the oil recovery profile in each core flooding test may differ depending on the heterogeneity and permeability of the rock samples. In this study, Berea sandstone samples are used with permeability ranging from 52.76 mD to 134.48 mD. Threfore, the main reason the oil recovery for 0.025 wt% TiO₂ combined with 1.25 wt% case shows a slight delay compared to other cases, likely due to the core having the lowest permeability.

## 4 Conclusion

We have investigated the performance of titanium dioxide nanoparticles and AEC surfactant in sandstone samples, as well as their relationship with incremental oil recovery. The investigation consists of one-dimensional core flooding tests with five different combinations of injected fluids. The results were carefully analyzed, complimented with additional data on interfacial tension, contact angle, and zeta potential, and we found that:

1. The addition of TiO_2_ nanoparticles to AEC surfactant can enhance its performance by lowering the interfacial tension (Megayanti et al., 2023) and shifting the water contact angle to a more water-wet condition compared with natural brine only.
2. The highest oil recovery on Berea sandstone was achieved by combining AEC surfactant 1.25 wt% with 0.025 wt% TiO_2_ nanoparticles. This result was attributed to the reduction of the interfacial tension and the stability of TiO_2_ on AEC surfactant.
3. The stability of TiO_2_ nanoparticles in AEC surfactant has become an essential parameter that must be considered before implementing it on a larger scale, as unstable nanoparticles can reduce the performance of combined solutions and yield inefficient displacement to recover oil in porous media.

The results from this work are crucial to enhancing our understanding of the mechanism by which the combination of TiO_2_ nanoparticles with AEC surfactant for increasing oil recovery in sandstone. Nevertheless, additional investigations related to different ions in brine compositions are necessary to understand the impact of complex ions on the performance of AEC surfactant combined with TiO_2_ nanoparticles.

## Data Availability

The raw data supporting the conclusions of this article will be made available by the authors, without undue reservation.
